# Judicial Discomfort over ‘Innovative’ Treatment for Adolescents with Gender Dysphoria

**DOI:** 10.1093/medlaw/fwac018

**Published:** 2022-07-13

**Authors:** Michelle M Taylor-Sands, Georgina Dimopoulos

**Affiliations:** Melbourne Law School, University of Melbourne, Carlton, VIC 3053, Australia; Honorary Fellow, Murdoch Children’s Research Institute, Parkville, Australia; Swinburne Law School, Swinburne University of Technology, Hawthorn, Australia

**Keywords:** Adolescent health, Gender dysphoria, *Gillick* competence, Innovative treatment, Welfare jurisdiction, Welfare of the child

## Abstract

Medical treatment for adolescents with gender dysphoria has attracted considerable attention in recent years, with continuing court involvement in Australia and recent judicial review proceedings in the UK. In *Re Imogen [No 6]*, the Family Court of Australia held that an application to the Family Court is mandatory if a parent or a medical practitioner of an adolescent diagnosed with gender dysphoria disputes the diagnosis, the adolescent’s capacity to consent, or the proposed treatment. In this article, we examine the Family Court’s rationale for preserving its welfare jurisdiction in gender dysphoria cases. We analyse case law developments in Australia and more recently in the UK and identify a thread of judicial discomfort in gender dysphoria jurisprudence about adolescents consenting to medical treatment that the court perceives to be ‘innovative’, ‘experimental’, ‘unique’, or ‘controversial’. We explore whether treatment for gender dysphoria can be characterised as ‘innovative’ and identify four factors that appear to be influencing courts in Australia and the UK. We also consider how such a characterisation might impact (if at all) on an adolescent’s capacity to consent to gender dysphoria treatment. We critique the ongoing role of courts in these cases and recommend a robust decision-making framework for gender dysphoria treatment to minimise court involvement in the future.

## I. INTRODUCTION

Medical treatment for adolescents with gender dysphoria has attracted considerable attention in recent years, with continuing involvement by the Family Court in Australia and recent judicial review proceedings in the UK.^1^ Although Australian and UK courts have not gone as far as some US legislatures, which have moved to criminalising gender-affirming care,^2^ commentators have criticised recent judicial involvement as ‘a step in the wrong direction’^3^ and ‘out of step with international norms’.^4^ In *Re Imogen (No 6)*,^5^ the Family Court of Australia held that where a parent of an adolescent diagnosed with gender dysphoria disputes the diagnosis, the adolescent’s capacity to consent or the proposed treatment, clinicians must seek court authorisation. The Court further held that, in cases where an adolescent is found to be *Gillick* competent but there remains a dispute about treatment, the Court must assess whether treatment is in the adolescent’s best interests. *Re Imogen* follows a line of judicial authority in Australia that has grappled with the scope of the welfare jurisdiction under section 67ZC of the Family Law Act 1975 (Cth) (Family Law Act) in gender dysphoria cases. We analyse case law in Australia and the UK over recent years and identify a thread of judicial discomfort in gender dysphoria jurisprudence about adolescents consenting to medical treatment that courts perceive to be ‘innovative’,^6^ ‘experimental’,^7^ ‘novel’,^8^ ‘unique’,^9^ or ‘controversial’.^10^

In Section II, we explain the legal and regulatory framework for the medical treatment of gender dysphoria in adolescents, including the welfare jurisdiction under section 67ZC of the Family Law Act, and the role of clinical treatment guidelines and standards. In Section III, we examine the Australian case law, which we argue exposes the Family Court’s concern to retain oversight of medical treatment for gender dysphoria in adolescents owing to the perceived ‘innovative’ nature of that treatment. The notion of ‘innovation’ in this context relates to treatment that lacks a clear or consistent line of medical authority, and for which minimal longitudinal data exist to support long-term efficacy. In Section IV, we examine how the notion of innovative treatment influenced the Family Court in *Re Imogen*. We argue that evidence of ‘emerging debate’^11^ and ‘alternate thinking’^12^ within the medical community triggered the Family Court’s reinstatement of a court authorisation requirement in cases involving a dispute over diagnosis, consent, or treatment. In Section V, we draw on recent UK case law and medico-legal literature to ascertain which aspects of gender dysphoria treatment have led courts to intervene in these cases. We argue that, even if treatment for gender dysphoria can be described as ‘innovative’, this should not impact on a competent adolescent’s capacity to consent to treatment. We conclude by evaluating the ongoing role of courts in cases involving treatment for gender dysphoria and their reluctance to engage with current clinical and ethical debate. We recommend a robust clinical decision-making framework for gender dysphoria treatment to minimise court involvement in the future. We argue elsewhere that courts should be a last resort for dealing with controversies over treatment as court processes can be psychologically burdensome, time-consuming, costly, and potentially harmful to adolescents and their family relationships.^13^

## II. THE REGULATORY FRAMEWORK FOR THE MEDICAL TREATMENT OF GENDER DYSPHORIA IN ADOLESCENTS

### A. Gender Dysphoria: Diagnosis and Treatment

Gender dysphoria is a medical condition characterised by a person experiencing distress when their ‘birth sex’ and self-perception of being male or female are misaligned, such that they feel ‘trapped’ in the wrong body.^14^ The current diagnostic manual used in Australia is the fifth edition of the American Psychiatric Association’s Diagnostic and Statistical Manual of Mental Disorders (DSM-5).^15^ For a child to be diagnosed with gender dysphoria, the DSM-5 requires the child to experience a ‘marked incongruence’ between their expressed or experienced gender and their gender assigned at birth, which persists for more than 6 months, and causes clinically significant distress or impairment in social, school or other important areas of functioning.^16^ For a diagnosis to be made, gender dysphoria must manifest in at least six of eight ways listed in the DSM-5, including ‘a strong desire to be of the other gender or an insistence that one is the other gender’, ‘a strong dislike of one’s sexual anatomy’, and ‘a strong desire for the physical sex characteristics that match one’s experienced gender’.^17^

Medical treatment for gender dysphoria in children and adolescents in Australia is guided by clinical treatment guidelines and standards of care. These include the World Professional Association for Transgender Health (WPATH) Standards of Care,^18^ the Endocrine Society Clinical Practice Guideline,^19^ and Australian Standards of Care and Treatment Guidelines for Trans and Gender Diverse Children and Adolescents (Australian Standards).^20^ These standards and clinical guidelines adopt a ‘gender-affirming’ model of care for transgender children and adolescents. A gender-affirming approach ‘holistically attends to transgender people’s physical, mental, and social health needs and well-being while respectfully affirming their gender identity’.^21^ Under this approach, accepted medical treatment occurs in two stages. The first stage involves the administration of puberty-suppressant hormones, known as ‘blockers’. The second stage of treatment involves the administration of either testosterone or oestrogen to facilitate the adolescent’s transition to their affirmed sex. Both in Australia and internationally, the ‘informed consent’ model for gender-affirming care has developed as an alternative assessment model.^22^ The model involves ‘a shared decision-making process between the patient and their treating clinician’, with mental health support provided to the patient where required, but not as a precondition to accessing treatment.^23^ Recently, the legality of the informed consent model as it applies to transgender children and adolescents in Australia has been challenged.^24^

### B. Family Court Authorisation of Medical Treatment under the Welfare Jurisdiction

The ‘default position’ under Australian law is that parents can decide upon medical treatment for their child who is not yet able to consent, ‘reflected through the prism of the child[]’s best interests’.^25^ Subject to any court order in force, parents have a ‘bundle of rights’,^26^ defined in the Family Law Act as ‘parental responsibility’: ‘[a]ll the duties, powers, responsibilities and authority which, by law, parents have in relation to children’.^27^ The Family Court of Australia has said that parental responsibility ‘… provides the time frame within which it can be assumed by others … that parents are empowered at law to make decisions for the protection and benefit of the child’.^28^

However, certain kinds of medical treatment, which have come to be known as ‘special medical procedures’, lie beyond the scope of parental responsibility and require court authorisation pursuant to section 67ZC of the Family Law Act. Section 67ZC provides that, in addition to the jurisdiction under part VII of the Act in relation to children, a court has jurisdiction to make orders relating to the welfare of children, having regard to the best interests of the child as the paramount consideration. The welfare jurisdiction under section 67ZC is considered to be ‘akin to’, although not limited to, the inherent *parens patriae* jurisdiction of State superior courts.^29^

Both parental responsibility and the welfare jurisdiction operate within a ‘best interests’ paradigm, although the power bestowed upon the court by section 67ZC is ‘broader than that of a parent or guardian’, given that ‘the Court is able to authorise action … which is beyond the scope of parental authority’.^30^ The Family Court of Australia has described the welfare jurisdiction under section 67ZC as ‘essentially supervisory of parental responsibility’.^31^ In *Re Alex*, discussed below, Nicholson CJ described the welfare jurisdiction as ‘protective’ and ‘paternalistic’, cautioning that ‘in modern thinking about children and young people [it] must be understood with regard to their rights’.^32^

Two factual issues determine whether decisions regarding a child’s medical treatment will be beyond the scope of parental responsibility and will require court authorisation: first, whether the child is competent to consent to treatment and secondly, whether the subject matter of the application to the court falls within the category of ‘special medical procedures’ to which a parent or guardian cannot consent.^33^ While the term ‘special medical procedure’ does not have a settled meaning or legislative definition,^34^ it has come to refer to medical treatment for children that has certain features or factors and so requires court authorisation pursuant to section 67ZC of the Family Law Act, as we explain below.

The decision of the High Court of Australia in *Secretary, Department of Health & Community Services v JWB & SMB* (*Marion’s case*)^35^ remains the seminal Australian authority on the scope of the welfare jurisdiction under section 67ZC of the Family Law Act. *Marion’s case* involved an application to authorise the sterilisation of a 14-year-old girl with severe intellectual disabilities. A majority of the High Court identified various ‘factors involved in a decision to authorise sterilisation’ that justified its conclusion that the decision to authorise such a medical procedure ‘should not come within the ordinary scope of parental power to consent to medical treatment’.^36^ These factors are: that the proposed treatment is ‘invasive’, ‘irreversible’, and ‘non-therapeutic’ (that is, not ‘appropriately carried out to treat some malfunction or disease’); the ‘significant risk of making the wrong decision’ about a child’s present or future capacity to consent, or about the best interests of a child who cannot consent; and the ‘particularly grave’ consequences of a wrong decision being made.^37^ Medical treatment that had the features identified by the High Court required Family Court authorisation, as a ‘procedural safeguard’.^38^

In the case of Re Alex,^39^ Nicholson CJ expanded the scope of the welfare jurisdiction under section 67ZC of the Family Law Act to medical treatment for gender dysphoria in children and adolescents. The application before the Family Court was a ‘novel’ one:^40^ a government department, as legal guardian of a 13-year-old young person (anonymised in the judgment as ‘Alex’), applied pursuant to section 67ZC for the authorisation of medical treatment for Alex, who had been diagnosed with ‘gender identity disorder’.^41^ The evidence before the court did not establish that Alex had the capacity to consent to the proposed treatment.^42^ Nicholson CJ was satisfied that the medical treatment plan proposed for Alex fell within the category of ‘special medical procedures’ requiring court authorisation, as developed in Marion’s case. His Honour referred to ‘significant risks’ that attached to

embarking on a process that will alter a child or young person who presents as physically of one sex in the direction of the opposite sex, even where the court is not asked to authorise surgery

and held that on the evidence, it could not be said that treatment for childhood gender dysphoria was ‘to cure a disease or correct some malfunction’.^43^

The Full Court of the Family Court has gradually relinquished the court’s role in gender dysphoria treatment for children and adolescents. In *Re Jamie*,^44^ the Full Court held that stage one treatment (puberty blockers) could no longer be considered a special medical procedure^45^; but came to the opposite conclusion in relation to stage two treatment (gender-affirming hormones), owing to its irreversible effects.^46^ The Full Court held that court authorisation for stage two treatment was appropriate, unless the child was *Gillick* competent (ie, a mature minor). Importantly, the Full Court held that it was the Family Court’s role to determine the question of *Gillick* competence.^47^ Four years later, the Full Court in *Re Kelvin*^48^ departed from its decision in *Re Jamie*. It concluded that an application to the Family Court to determine a child’s *Gillick* competence was no longer mandatory where the child consented to stage two treatment, the child’s treating medical practitioners agreed that the child was *Gillick* competent, and the parents did not object to the treatment.^49^ However, the majority imposed a caveat: there was ‘no doubt’ that the Family Court had the jurisdiction and power to address questions about the need for court authorisation in circumstances of ‘genuine dispute or controversy’ between the child’s parents and treating medical practitioners about whether medical treatment for gender dysphoria should be administered.^50^ Similarly, the minority held that the Family Court had no role to play in relation to stage two treatment ‘unless there is a dispute about consent or treatment’.^51^ The majority also observed that

treatment that might not meet the description of having ‘grave or irreversible consequences’ might nevertheless fall outside of the scope of parental authority because of its novelty, or its experimental nature, or its place outside of accepted medical science …^52^

Having outlined how the welfare jurisdiction under section 67ZC of the Family Law Act evolved to capture medical treatment for children and adolescents with gender dysphoria, in the next section, we focus on the judicial characterisation of gender dysphoria treatment as ‘novel’, ‘experimental’, or falling ‘outside of accepted medical science’.

## III. ONGOING FAMILY COURT OVERSIGHT OF MEDICAL TREATMENT FOR ADOLESCENTS WITH GENDER DYSPHORIA

Although treatment for gender dysphoria is no longer considered a ‘special medical procedure’ in Australia, the Family Court has continued its oversight, which seems to be based—at least in part—on the perceived ‘innovative’ nature of this treatment.^53^ It is important to distinguish between the perceived ‘novelty’ of gender dysphoria as a medical condition, and the novelty of the proposed treatment. In *Re Jamie*, Bryant CJ observed that

the novelty of the condition described … [in *Re Alex*] is no longer supportable. The cases since *Re Alex* … would indicate that the condition is not as unusual as it presented itself in 2003 when *Re Alex* was decided.^54^

Family Court judges have recognised that the diagnosis of children with gender dysphoria in Australia has grown significantly over the past 15 years.^55^

However, the Family Court has emphasised on several occasions that the kinds of medical treatment that may fall within the court’s welfare jurisdiction will be influenced by all the circumstances surrounding the treatment, including developments in medical science. In *Re Alex*, Nicholson CJ noted that ‘[t]he categories of cases in which the welfare jurisdiction properly ought to be invoked are not closed’, particularly as ‘the march of science overtakes the perimeters of the settled law’.^56^ In *Re Sean and Russell*,^57^ Murphy J considered that it was ‘not possible, nor … desirable, to further define or list those procedures, treatments or the like which require court authorisation’.^58^

According to Bryant CJ in *Re Jamie*, the mere ‘possibility of different treatments’ and ‘different views about what treatment should be given—for example, whether a condition might be treated with medication or surgery, and which medications might be more effective than others’ would not, on that basis alone, render the treatment a special medical procedure.^59^ However, her Honour agreed that cases may arise involving issues that require court authorisation ‘due to the evolving state of medical knowledge’.^60^ The majority in *Re Kelvin* reinforced these earlier judicial views, observing that

the types of medical treatment for which court authorisation is required are neither closed nor confined to sterilization of a child who is not, and never will be, *Gillick* competent. Rather, as a general rule, whether court authorisation is required will be dependent upon the entirety of the circumstances surrounding the particular treatment.^61^

### A. Judicial Understandings of Medical Treatment for Gender Dysphoria as ‘Innovative’

The Australian gender dysphoria jurisprudence to date exposes what we argue is a judicial discomfort about adolescents consenting to medical treatment that the Family Court perceives to be ‘innovative’. We embrace the notion of ‘innovation’ in this context as capturing treatment that lacks a clear or consistent line of medical authority, and for which minimal longitudinal data exist to support long-term efficacy. This has manifested in gender dysphoria proceedings through a dearth of, or the contested nature of, expert or scientific evidence regarding the nature, risks, and long-term impacts of treatment.

The Family Court in gender dysphoria proceedings has displayed a consistently strong deference to the evidence of the medical practitioners of the child or adolescent seeking access to treatment. In *Re Bernadette*,^62^ for instance, the Family Court was asked to re-consider Re Alex and to determine whether parents have the authority to lawfully authorise stage one and/or stage two treatment without a court order.^63^ Collier J was not satisfied that the evidence established that there had been ‘such a change in the state of medical knowledge’ that would enable the court to disregard the views of the High Court in *Marion’s case* and of the Family Court in *Re Alex*.^64^ His Honour cited the absence of ‘clear-cut’ medical authority on the cause of gender dysphoria,^65^ and ‘considerable difference between what might be described as the British school and the Dutch school’ of thinking in relation to aspects of proposed treatment.^66^ In justifying the conclusion that medical treatment for gender dysphoria did not fall within the parameters of parental responsibility, Collier J in *Re Bernadette* observed that

… there still remains grave dispute within the medical community as to the best treatment that can be offered. I am satisfied that until there is a clear cut line of authority within the medical profession, it would be difficult for parents to reach an informed conclusion in every case.^67^

In subsequent gender dysphoria cases, the Family Court has referred to ‘uncertainties surrounding the treatment’^68^ and ‘significant controversy within the scientific community’^69^ regarding the risks and potentially adverse effects of gender-affirming hormone treatment that ‘is not well-understood’.^70^ The Court has noted ‘limited longitudinal research on the impact of long term cross hormone treatment’,^71^ and observed that ‘the long-term effects of the proposed treatment are still being studied’.^72^ It has also referred to ‘no evidence one way or the other’ in relation to a particular risk of the treatment, given that it ‘had not been measured by any long-term outcome study’.^73^ The absence of consensus within the medical community, coupled with the long-term impacts of gender-affirming hormone treatment as ‘an area requiring ongoing research’,^74^ we suggest has justified the Family Court’s ongoing role in the medical treatment process for gender dysphoria in children and adolescents.

However, the Full Court in *Re Kelvin* displayed an appreciation of the need for ‘the law … to effectively reflect the current state of medical knowledge’.^75^ The majority noted that in ‘each and every case’ in which court authorisation had been sought for an adolescent’s treatment for gender dysphoria, the decision had been informed by ‘comprehensive evidence from a miscellany of medical specialists from different disciplines (for example, psychiatry, psychology, paediatrics, and endocrinology) …’.^76^ The Full Court also observed that ‘over time the expert evidence adduced in [gender dysphoria] … cases reflected advances in medicine’.^77^

### B. Judicial Appreciation of ‘Advances in Medical Science’ and the Evolving State of Medical Knowledge

In *Re Kelvin*, outlined in Section II.B above, the issue for the Full Court's determination was whether gender dysphoria should continue to require the ‘filter’ of court involvement, ‘when no such filter is required in most cases involving other medical conditions’.^78^ The majority situated its decision within the historical trajectory of the welfare jurisdiction in gender dysphoria proceedings. Nicholson CJ in Re Alex, in imposing a court authorisation requirement for both stage one and stage two treatments, alluded to unsettled medical authority in relation to the diagnosis of and treatment for gender dysphoria. His Honour observed that ‘[t]he aetiology of a compelling desire to make the transition to become the opposite sex has not been definitively established’;^79^ and that ‘[t]he current state of knowledge would not … enable a finding that the treatment would clearly be for a “malfunction” or “disease” and thereby not within the jurisdiction of this Court.’^80^ The Full Court in *Re Jamie* had departed from *Re Alex* in finding that stage one treatment was ‘therapeutic’ and fully reversible, and so fell within the wide ambit of parental responsibility, because ‘not only the state of medical science had moved on, but the Court’s understanding of the same had evolved’.^81^ The majority in *Re Kelvin* also observed that an assessment of whether a particular treatment is therapeutic or non-therapeutic (on the distinction drawn in *Marion’s Case*) must depend on, among other things, ‘evolving medical science which, notoriously, occurs at a very rapid pace’.^82^

The majority in *Re Kelvin* conceded that judicial understandings of gender dysphoria and its treatment had ‘fallen behind’ advances in medical science.^83^ It referred to the release of a new edition of the DSM-5 between the Full Court’s hearing of *Re Jamie* and *Re Kelvin*;^84^ the development of international and Australian standards of care for the treatment of gender dysphoria in adolescents; and increased knowledge of the risks associated with not providing treatment.^85^ According to the majority, there was ‘no question that the state of medical knowledge has evolved since the decision in *Re Jamie*’.^86^ The majority identified ‘legally relevant factual differences’ to justify its conclusion that it was ‘unnecessary and indeed inappropriate’ for the Full Court to find that *Re Jamie* was ‘plainly wrong’.^87^ These differences included

the advances in medical science regarding the purpose for which the treatment is provided, the nature of the treatment, and the risks involved in undergoing, withholding or delaying treatment.^88^

Notwithstanding its divergent reasoning to arrive at the same conclusion, the minority observed that ‘over time the expert evidence adduced in [gender dysphoria] … cases reflected … advances in medicine’.^89^

The Full Court in *Re Kelvin* thus acknowledged that the law had fallen out of step with medical and scientific progress in the treatment of gender dysphoria—progress which mandated a conclusion that not only the condition, but also treatment for that condition, was no longer ‘novel’ or ‘uncertain’ enough as to require court authorisation. Importantly, however, the majority in *Re Kelvin* emphasised that the Full Court’s focus was upon whether there was any role for the Family Court in cases lacking a dimension of controversy or dispute

where there is no dispute between parents of a child who has been diagnosed with Gender Dysphoria, and where there is also no dispute between the parents and the medical experts who propose the child undertake treatment for that dysphoria.^90^

The Family Court in *Re Imogen* embraced this notion of ‘controversy’ over *Gillick* competence, diagnosis or treatment, to reaffirm the court’s role in the medical treatment of gender dysphoria in certain circumstances.

## IV. *RE IMOGEN*: ‘EMERGING DEBATE’ AND THE FAMILY COURT’S ROLE IN DISPUTES ABOUT CONSENT, DIAGNOSIS, OR TREATMENT OF GENDER DYSPHORIA

### A. An Overview of the Re Imogen Proceedings

‘Imogen’, as she was anonymised in the judgments, had been diagnosed with gender dysphoria and assessed as *Gillick* competent by her treating medical practitioners. Imogen was 16 years of age at the time of her father’s application to the Family Court. She had been undertaking stage one treatment, and had been expressing ‘a consistent, persistent, and insistent’ wish to progress to stage two treatment for almost 2 years.^91^ However, Imogen’s mother disputed Imogen’s diagnosis and the finding of *Gillick* competence and did not consent to Imogen commencing stage two treatment.^92^

The trial judge, Watts J, identified four questions that Imogen’s case raised about the current law for children and adolescents diagnosed with gender dysphoria where there is a dispute about consent or treatment. These questions concerned whether an application to the Family Court is mandatory; whether a finding of *Gillick* competence would enable the adolescent to make their own treatment decisions; the nature of any order that should be made about *Gillick* competence; and the nature of the order that should be made if the adolescent’s consent is insufficient.^93^

Watts J’s answers to these questions reaffirmed the Family Court’s role in gender dysphoria treatment for children and adolescents in circumstances of dispute or controversy.^94^ His Honour concluded that an application to the Family Court is mandatory if a parent or a medical practitioner of an adolescent diagnosed with gender dysphoria disputes the diagnosis, the adolescent’s *Gillick* competence, or the proposed treatment.^95^ Once an application to the Family Court is made (whether or not mandatory), Watts J held that the Court should make a finding about *Gillick* competence. If the dispute concerns *Gillick* competence only, the Court should determine whether the adolescent is *Gillick* competent by way of a declaration under section 34(1) of the Family Law Act, without the need for a determination based on ‘best interests’ considerations. A declaration of *Gillick* competence determines the dispute and the adolescent can make a decision about their treatment without court authorisation.^96^

However, where a dispute concerns diagnosis or treatment, Watts J concluded that a finding of *Gillick* competence is not determinative. Notwithstanding a finding of *Gillick* competence, the Family Court should determine the diagnosis; determine whether treatment is appropriate, following a best interests’ assessment; and make an order authorising or not authorising treatment pursuant to section 67ZC of the Family Law Act.^97^ Watts J also held that a medical practitioner should not administer treatment without court authorisation in circumstances where an adolescent seeks treatment but the adolescent’s parent or legal guardian does not consent.^98^

Watts J observed that the *Re Imogen* proceedings were heard ‘in the context of an emerging debate about the diagnosis and treatment’ of gender dysphoria.^99^ In the next section, we focus upon the expert evidence put before the Family Court in *Re Imogen*, which was said to demonstrate ‘the emergence of alternate thinking’ in relation to the treatment of gender dysphoria and the long-term impacts of that treatment.^100^ We argue that such evidence raised the spectre of dispute within the medical community, which was not before the Full Court in *Re Kelvin*, yet which purportedly warranted the Family Court’s ongoing protective oversight role.

### B. Evidence of ‘The Emergence of Alternate Thinking’ about Gender Dysphoria Treatment

As noted in Section III.B above, the Full Court in *Re Kelvin* removed the Family Court from the medical treatment process for gender dysphoria in adolescents, in light of ‘advances in medical science in treating and understanding’ gender dysphoria.^101^ In *Re Imogen*, the Australian Human Rights Commission, an intervener in the proceedings, argued that statements of legal principle in *Re Jamie* must be viewed in light of the ‘difference in the state of medical knowledge’ between when that case was decided at first instance in 2011, and when *Re Kelvin* was decided in 2017—and in particular, the ‘changed understanding about the nature of treatment’.^102^ Watts J rejected this argument. According to his Honour, a ‘logical extension’ of that argument would require

a consideration of the volume of evidence in this case which demonstrates a proliferation of academic and other writings since *Re Kelvin* and the emergence of alternate thinking about treatment and questions arising from the state of knowledge in respect of the long-term implications of current medical treatment for Gender Dysphoria.^103^

Such ‘alternate thinking’ and clinical questions emanating from the state of knowledge about the long-term effects of gender dysphoria treatment for adolescents emerged from the expert evidence in *Re Imogen*. In *Re Kelvin*, the majority had observed that in no stage two gender dysphoria case had contradictory evidence been forthcoming to challenge the desirability of the relevant medical treatment.^104^ The *Re Imogen* proceedings offered that contradictory evidence. Medical evidence was provided by Imogen’s treating psychiatrist and treating endocrinologist (called by Imogen’s father), a psychiatrist (called by Imogen’s mother), and an academic with a background in therapy (called by the independent children’s lawyer). Watts J noted that two of these experts adopted ‘fundamentally different diagnostic frameworks, methods, and conceptualisation of the experience’ of gender dysphoria.^105^ The definition of gender dysphoria and the stages of gender-affirming treatment as described in the Australian Standards were not in issue.^106^ Rather, the point of contention lay in the efficacy of the proposed treatment for Imogen, namely, the administration of gender-affirming hormones.

The ‘orthodox middle’, according to Watts J, was the Australian Standards, which adopt a ‘multi-disciplinary approach’ to treatment using gender-affirming hormones that is ‘currently accepted by the majority of the medical profession’ and was followed by Imogen’s treating medical practitioners.^107^ This is the gender-affirming approach outlined in Section II.A above. Watts J made two pointed observations about the Australian Standards. First, his Honour found that they did not accurately reflect the current state of the law in circumstances where there is a dispute about treatment.^108^ Secondly, Watts J noted that while the Australian Standards ‘assert that they are based upon available empirical evidence and clinical consensus’, they also acknowledged that future research was warranted and was ‘likely to influence future recommendations’.^109^

The mother’s adversarial expert psychiatrist advocated a ‘more conservative’ and ‘alternative’ approach.^110^ He suggested that psychotherapy, rather than medication, should be the preferred treatment method for gender dysphoria in adolescents. Watts J accepted that this was a ‘risky and unproven strategy’.^111^ His Honour also referred to a ‘less conservative approach’, being the ‘informed consent’ model made available through particular general practitioners, who prescribed gender-affirming hormones to 16- and 17-year-olds ‘without knowing whether their parents or legal guardians dispute whether that treatment should be prescribed’.^112^ Two experts gave contrasting evidence regarding the acceptance and practice of this informed consent model in Australia: according to one, the model was being adopted by ‘an increasing number of medical practitioners’, while the other had ‘not seen any evidence that “informed consent” is becoming a widely accepted model of treatment’.^113^

Watts J observed that research literature in transgender health had ‘expanded rapidly’ in the last decade, particularly since the *Re Kelvin* decision.^114^ His Honour identified ‘different views’ among the experts in the case about the state of current research into the diagnosis and treatment of gender dysphoria.^115^ A particular point of disagreement was the research base for the gender-affirming care model. The mother’s expert expressed concern about ‘the lack of adequate study into the physical and psychological long-term effects of hormonal and surgical interventions’.^116^ Watts J noted that both the Australian Standards and the academic expert in the case acknowledged the need for further research into the long-term outcomes of current treatments under the gender-affirming care model.^117^ His Honour highlighted parts of a letter from the Royal Australian College of Physicians (RACP) to the Federal Minister for Health, who had sought advice on the treatment of gender dysphoria in children and adolescents in Australia. That letter recommended that patients and families ‘be provided with information about the limitations of the available evidence’ concerning gender dysphoria.^118^ Relevantly, it described gender dysphoria treatment as

an emerging area of healthcare where existing evidence on health and wellbeing outcomes of clinical care is limited due to the relatively small number of studies, the small size of study populations, the absence of long-term follow up and the ethical challenges of robust evaluation when control (no treatment) is not acceptable.^119^

The Family Court’s concern about ‘emerging debate’ within the medical community, and gaps in the research concerning gender dysphoria treatment, are neatly captured by Imogen’s ‘measured response’ to a question put to her in an assessment by the mother’s expert: ‘we just don’t know’.^120^ The many perceived unknowns—including why transgender patients are lost to follow-up, the exponential rise in gender dysphoria cases in the past decade, issues of regret and de-transitioning, and the surge in adolescents identifying as transgender without a reported childhood history^121^—we suggest prompted the Family Court in *Re Imogen* to mandate court involvement in cases involving a dispute about diagnosis, *Gillick* competence, or treatment.

Watts J quoted from the majority judgment in *Re Kelvin* that while ‘routine treatments for everyday medical conditions’ do not require court authorisation, some circumstances ‘may dictate the need for court intervention’—including, relevantly, ‘disputes between parents’ or ‘experimental or novel treatment or treatment for unusual or novel conditions’.^122^ The *Re Imogen* judgment emphasised the circumstance of ‘disputes between parents’ to justify the Family Court’s ongoing involvement in gender dysphoria treatment.^123^ This was a case involving parents in ‘warring camps’,^124^ and Watts J sought to resolve a ‘controversy’ about what the leading Full Court authorities—*Re Jamie* and *Re Kelvin—*decided about ‘cases where there is dispute about consent or treatment’.^125^ Watts J reflected that the evidence about Imogen sourcing unprescribed medication from overseas was ‘troubling but spoke eloquently of the dangers that have been created by the dispute in this case’.^126^ Our analysis in this section has served to show that the nature of the treatment for gender dysphoria was of greater concern to the Court than the fact of dispute, given that the medical and scientific evidence is not yet well established or agreed upon.

## V. IS TREATMENT FOR GENDER DYSPHORIA ‘INNOVATIVE’ AND HOW MIGHT THIS IMPACT CAPACITY TO CONSENT?

The analysis of Re Imogen in Section IV above reveals ongoing judicial concern over protecting adolescents diagnosed with gender dysphoria from making decisions about their own bodies and identities, in relation to treatment the court (still) perceives to be innovative or novel in nature. While the term ‘innovative treatment’ has not been used explicitly by the Family Court in gender dysphoria cases,^127^ the Court has referred variously to ‘experimental’ or ‘novel’ treatment,^128^ medical evidence that is not ‘clear cut’,^129^ and ‘evolving medical science, which, notoriously, occurs at a very rapid pace’.^130^ The notion of ‘innovation’ in this context appears to relate to treatment that lacks a clear or consistent line of medical authority, and for which minimal longitudinal data exist to support long-term efficacy, in part due to the ethical inability to conduct randomised control trials.^131^

In this section, we compare the judicial discomfort around gender dysphoria treatment in Australia with that in the UK, where recently the courts have expressly articulated concern in relation to the use of puberty blockers as stage one of the medical treatment process. UK courts have highlighted certain aspects of gender dysphoria treatment as justifying court involvement in what they perceive to be ‘unique’ or ‘controversial’ cases.^132^ We identify four factors that appear to be influencing Australian and UK courts’ characterisation of treatment for gender dysphoria as a special category that warrants court oversight. We also examine the notion of ‘innovative’ treatment as defined in the medico-legal and ethics literature and draw some common threads with the judicial commentary on treatment for gender dysphoria. We argue that, even if treatment for gender dysphoria can be described as ‘innovative’, this should not impact on a competent adolescent’s capacity to consent to treatment.

### A. Recent UK Jurisprudence on Gender Dysphoria Treatment

Judicial discomfort around what courts perceive to be ‘innovative’ treatment was clearly articulated by the UK High Court in *Bell v Tavistock*.^133^ This case involved an application for judicial review and declarations that the UK Gender Identity Development Service (GIDS) acted unlawfully in prescribing puberty blockers to children under the age of 18 years. The High Court did not find that GIDS acted unlawfully, but made declarations that undermined the capacity of adolescents to consent to treatment with puberty blockers and suggested that court authorisation may be needed to administer puberty blockers in future cases. The High Court described the administration of puberty blockers as ‘a very unusual treatment’ for several reasons:

Firstly, there is real uncertainty over the short and long-term consequences of the treatment with very limited evidence as to its efficacy, or indeed quite what it is seeking to achieve. This means it is, in our view, properly described as experimental treatment. Secondly, there is a lack of clarity over the purpose of the treatment: in particular, whether it provides a ‘pause to think’ in a ‘hormone neutral’ state or is a treatment to limit the effects of puberty, and thus the need for greater surgical and chemical intervention later, as referred to in the Health Research Authority report. Thirdly, the consequences of the treatment are highly complex and potentially lifelong and life changing in the most fundamental way imaginable. The treatment goes to the heart of an individual’s identity, and is thus, quite possibly, unique as a medical treatment.^134^

The High Court in *Bell* observed that ‘the clinical intervention we are concerned with here is different in kind to other treatments or clinical interventions’.^135^ According to Dunne, the High Court’s ‘scepticism’ about the efficacy of puberty blockers contributed to its determination that such a treatment is ‘experimental’, as the High Court did not engage with the ‘growing body of international scholarship’ on the benefits of early medical intervention for gender dysphoria in adolescents.^136^ In the subsequent case of *AB v CD*,^137^ the High Court similarly acknowledged that puberty blockers have ‘life-changing and life-long consequences, the implications of which are not fully understood’, and clinical and ethical views on their use differ significantly.^138^

On appeal, the Court of Appeal in *Bell v Tavistock* described the treatment of gender dysphoria as ‘controversial’, but held that it was a question of fact as to whether an adolescent can consent to treatment with puberty blockers.^139^ The following comments highlight a judicial reluctance to engage with the broader clinical, moral, and ethical debates surrounding gender dysphoria treatment:

Medical opinion is far from unanimous about the wisdom of embarking on treatment before adulthood. The question raises not only clinical medical issues but also moral and ethical issues, all of which are the subject of intense professional and public debate. Such debate, when it spills into legal proceedings, is apt to obscure the role of the courts in deciding discrete legal issues.^140^

Although the decision by the Court of Appeal has been welcomed as a ‘huge win’^141^ and a ‘positive step forward’^142^ for trans people and their families, others have emphasised that the court was careful not to take a position on the debate over puberty blockers.^143^ Some commentators have expressed concern that ‘legal judgments which interfere with necessary medical treatment for transgender youth, undertaken in a shared decision-making process between patients and qualified clinicians’, have the potential to harm the children and adolescents seeking treatment.^144^ In recognising the ongoing controversy in this area and the potential for civil action against clinicians in individual cases, the Court of Appeal highlighted the potential for courts to be involved in future gender dysphoria cases.^145^

### B. Factors Influencing Courts in Categorising Treatment for Gender Dysphoria as ‘Innovative’

From our review and analysis of the Australian and UK case law on gender dysphoria treatment for children and adolescents, we have identified four key factors underpinning the courts’ cautious approach, and the finding that treatment for gender dysphoria is, variously, ‘innovative’, ‘experimental’, ‘novel’, ‘unique’, or ‘controversial’. This finding has led to judicial concerns about the impacts of treatment on an adolescent’s future autonomy and ability to assess the benefits and risks, compelling courts to protect adolescents from their own decision-making.^146^ These concerns expose a tension between best interests and autonomy, and challenge the capacity of adolescents to make decisions that will affect their future choices, yet which they may in the present not fully understand.^147^ In *Bell v Tavistock*, the High Court found that:

For many children, … it will not be possible to conceptualise what not being able to give birth to children (or conceive children with their own sperm) would mean in adult life. Similarly, the meaning of sexual fulfilment, and what the implications of treatment may be for this in the future, will be impossible for many children to comprehend.^148^

The High Court considered that there was ‘no age appropriate way to explain to many of these children what losing their fertility or full sexual function may mean to them in later years’.^149^ However, protecting a child’s right to an ‘open future’^150^ in the context of gender dysphoria treatment is complicated because, as the Court of Appeal in *Bell v Tavistock* recognised, ‘neither puberty suppression nor allowing puberty to occur can be regarded as a neutral act’.^151^

The first factor relied on by Australian and UK courts to justify a cautious approach in gender dysphoria cases is the disharmony within the medical community in relation to gender dysphoria treatment. The Family Court in *Re Imogen* emphasised the ‘emergence of alternate thinking’ and ‘emerging debate’ within the medical community about the long-term effects of gender dysphoria treatment to mandate court involvement in cases involving a dispute.^152^ In *AB v CD*, the High Court of England and Wales noted that the use of puberty blockers ‘raises unique and highly controversial issues’ and described the division of clinical and ethical views as ‘highly polarised’.^153^ However, the High Court in that case argued that these matters should be addressed ‘in a regulatory and academic setting’,^154^ rather than via court oversight.

Secondly, and related to the first factor, is uncertainty over short- and long-term consequences of treatment, given the limited longitudinal evidence about efficacy and risks.^155^ Potential risks associated with stage one hormone treatment for gender dysphoria (puberty blockers) include reduced bone density, genital atrophy (which may compromise future genital reconstructive surgery), and negative cognitive and psychosocial impacts associated with delayed puberty.^156^ Risks of stage two treatment (gender-affirming hormones) include permanent infertility, which may impact on the adolescent’s future reproductive choices.^157^ Medical opinion on the nature and significance of the various risks associated with gender dysphoria treatment for adolescents varies. Duffy argues that puberty blockers have been shown internationally to be ‘both safe and reversible’,^158^ whereas the Royal Australian and New Zealand College of Psychiatrists states that ‘there is a paucity of quality evidence’ on patient outcomes for gender dysphoria, particularly for children and adolescents.^159^ There is growing clinical consensus that the benefits of hormone and surgical treatment outweigh the risks. However, some experts emphasise the absence of randomised controlled trials in this field and note that current ‘[r]ecommendations are based on low level evidence, broad and open to interpretation’.^160^ Others argue that there is a ‘moral obligation to scientifically study’ the impacts of medical treatment for gender dysphoria on the well-being and developing autonomy of children and adolescents.^161^ It is also important to balance the risks associated with treatment for gender dysphoria with the risks associated with not providing treatment.^162^ These include increasing depression, anxiety and suicidality, social withdrawal, illegally accessing medications (as occurred in *Re Imogen*), and eating disorders.^163^

The third factor that appears to be influencing Australian and UK courts in treating gender dysphoria cases as a special category is a perceived lack of clarity over the purpose of the treatment, and disagreement about the aetiology of the condition being treated. The High Court in *Bell v Tavistock* highlighted its concern about the ‘direct physical consequences’ of treating a condition that ‘has no direct physical manifestation’.^164^ The High Court also queried the categorisation in the DSM-5 of gender dysphoria as a psychological condition, noting that

in other cases, medical treatment is used to remedy, or alleviate the symptoms of, a diagnosed physical or mental condition, and the effects of that treatment are direct and usually apparent. The position in relation to puberty blockers would not seem to reflect that description.^165^

Horowicz challenges the need to pathologise gender diversity to promote access to treatment for gender dysphoria.^166^ He notes that the different ways in which healthcare systems provide gender dysphoria services, and the way in which different societies and cultures view gender diversity, have created barriers to accessing appropriate treatment.^167^ These concerns reflect Brunskell-Evans’ view that the ‘transgender child’ is a category constructed by medico-legal discourse.^168^ Our analysis reinforces these concerns: while medical treatment for gender dysphoria in Australia no longer falls within the ‘special medical procedure’ category requiring court authorisation, the Family Court remains reluctant to ‘let go’, in part due to its trepidation over the treatment sought.

Fourthly, courts highlight the fact that consequences of gender dysphoria treatment are complex and potentially life-changing.^169^ The Family Court in *Re Imogen* was influenced by clinical uncertainty about the long-term effects of gender dysphoria treatment for adolescents, which emerged from the expert evidence.^170^ Although *Re Imogen* focused on stage two gender-affirming hormone treatment, the High Court in *Bell v Tavistock* held that the consequences that flow from stage one puberty blocking treatment include those that follow on from progression to stage two treatment.^171^ Moreton describes this conclusion as ‘problematic’ and inconsistent with Australian jurisprudence, which previously categorised stage one treatment as a separate and distinct stage to stage two treatment, to which a competent adolescent could consent without court approval.^172^ The Court of Appeal in *Bell v Tavistock* also emphasised the need for clinicians to take ‘great care’ in prescribing puberty blockers and cross sex hormones ‘in the light of evolving research and understanding of the implications and long-term consequences of such treatment’ to avoid regulatory or civil action.^173^

The four factors just described appear to have influenced the Australian and UK courts in taking a cautious approach to cases involving treatment for gender dysphoria. However, it is important to highlight that the concept of ‘innovative’ treatment more broadly is underdeveloped in case law. There is some precedent in UK law for courts taking a different approach to experimental treatment.^174^ In *Simms v Simms*, Butler-Sloss J described the treatment of Creutzfeldt–Jakob disease with the drug Pentosan Polysulphate as ‘experimental treatment with unknown risks and benefits’, but held that administration of the treatment was justified in the circumstances without court approval.^175^ In *AB v CD*, Lieven J distinguished treatment with puberty blockers from *Simms v Simms*, which involved a fatal condition with no alternative treatment. She argued that a more cautious approach may well be justified in relation to puberty blocker treatment, as the ‘factual, clinical and ethical issues’ surrounding such treatment are ‘different’.^176^

In *R (Burke) v General Medical Council*, Munby J distinguished between routine medical practice and ‘innovative, experimental or untested forms of treatment’.^177^ In the more recent case of *B v D*, Baker J described stem cell treatment for an adult who lacks capacity due to a traumatic brain injury as ‘new’ on the basis

that it is unsupported by any or at least any significant body of research, that it has not been subjected to clinical trials, and that the evidence that it is, or might be, an effective treatment for traumatic brain injury is almost entirely anecdotal.^178^

At least some of these cases appear to describe treatments that have less clinical certainty than current treatment for gender dysphoria, although there is no clear threshold outlined by the court as to where the line should be drawn.

### C. Attempts to Define ‘Innovative’ Treatment in Medico-Legal and Ethics Literature

Definitions in the medico-legal and ethics literature on medical ‘innovation’ vary. However, as with the judicial commentary discussed above, there are some common threads that can be drawn. The conceptualisation of certain treatments as ‘innovative’ has been discussed in medical literature for over 50 years.^179^ In 1974, the US National Commission for the Protection of Human Subjects of Biomedical and Behavioral Research distinguished ‘innovative practice’ from both ordinary clinical practice and clinical research.^180^ Specifically, the Commission found that a procedure that is ‘experimental’ insofar as it is ‘new, untested or different’ does not automatically place it in the category of research.^181^ According to Earl, this view suggests that ‘therapeutic, preventive, or diagnostic (i.e. clinical) interventions are innovative if they deviate from standard or accepted clinical practice’.^182^ Recognising the potential for ambiguity in this definition, Earl more recently defined ‘innovative practice’, drawing on ethics literature, as a deviation from ‘the idealized expert-consensus standard’, which is ‘best supported by the available scientific evidence, clinical experience, and expert judgment’.^183^ This definition focuses on evidence base and expert-consensus.

Commentators from different disciplines have attempted to define ‘innovative treatment’ based on specific factors. For example, Brierley and Larcher focus on clinical uncertainties around efficacy and side-effects of treatment by describing innovative therapy as

any newly introduced treatment, or a new modification to an existing therapy with unproven efficacy and side effect profile, which is being used in the best interests of a patient, often on an experimental and/or compassionate basis.^184^

Other definitions refer to treatment that is ‘less likely to accord with *accepted* practice’,^185^ and observe that the adjective ‘innovative’ ‘informs patients that the suggested treatment is new and not standard’, which may be ‘linguistically significant’, because it ‘sounds positive in comparison to describing a treatment as “unproven” or “untested”’.^186^

McHale accepts that what may amount to an ‘innovative’ treatment is ‘relative and a question of time and space’; ‘something which is unique to this context and has never been used before in another clinical setting, or something which has been adapted from use in a different clinical setting’.^187^ She also appreciates that ‘definitional uncertainties’ concerning such treatment create a ‘practical problem in terms of ensuring effective oversight and subsequent accountability’.^188^ These issues of effective oversight and accountability indeed emerge from the gender dysphoria jurisprudence in both Australia and the UK, and the courts’ reluctance in Australia to cede their role in the medical treatment process for children and adolescents.

There is no clear consensus in the relevant literature as to whether treatment for gender dysphoria is ‘innovative’. O’Connor and Madden argue that gender-affirming hormone treatment remains to some extent ‘experimental’, as doubts linger about whether sufficiently well-designed studies exist in relation to the long-term efficacy of that treatment.^189^ In contrast, Moreton rejects the categorisation of stage one treatment for gender dysphoria as ‘experimental’, noting that puberty blockers are safe and reversible, and that most drugs have unknown long-term effects.^190^

The common threads in the literature on ‘innovative treatment’ raise similar issues to those in the judicial commentary on treatment for gender dysphoria. A dominant concern is clinicians’ potential liability in negligence for adverse outcomes following innovative treatment, or for failing to obtain the patient’s valid consent to treatment, or for failing to discharge their duty to inform the patient of the risks.^191^ Cockburn and Fay argue that the potential unknown risks of innovative treatment may impact on the legal obligations of clinicians in terms of their duty to inform patients of ‘material risks, particularly communicating uncertainty and the possibility of unknown risks; potential conflicts of interests…; and the likelihood of optimism bias in decision making’.^192^

These threads also reflect, to a lesser degree, some of the ‘features’ or ‘factors’ that the High Court identified in *Marion’s case* for ‘special medical procedures’ requiring court authorisation. As we outlined in Section II.B above, these factors are: that the proposed treatment is ‘invasive’, ‘irreversible’, and ‘non-therapeutic’; the ‘significant risk of making the wrong decision’ in relation to a child’s present or future capacity to consent, or about the best interests of a child who cannot consent; and the ‘particularly grave’ consequences of a wrong decision being made.^193^ Although treatment for gender dysphoria is no longer considered a ‘special medical procedure’ on the basis that it is now recognised as ‘therapeutic’,^194^ the caution exercised by Australian and UK courts in gender dysphoria cases suggests that the presence of certain factors will lead to continued court involvement and oversight in some cases. In the final section, we consider whether classifying gender dysphoria treatment for children and adolescents as ‘innovative’ should impact on the consent process.

### D. Impacts on Capacity to Consent

Kimberley and others state that ‘innovative treatment options’ for gender dysphoria raise ethical challenges for the consent process, given that ‘for novel or innovative therapies … risks and benefits are not well documented or understood’.^195^ The potential unknown risks of innovative treatment and the challenges in adequately conveying these risks to adolescents appear to have a significant impact on the courts, even where an adolescent is found to be *Gillick* competent. According to the decision of the House of Lords in *Gillick*, an adolescent can consent to treatment when they have achieved ‘a sufficient understanding and intelligence to enable him or her to understand *fully* what is proposed’.^196^ The House of Lords in *Gillick* did not restrict the kinds of ‘matters’ that a child might be competent to decide to the facts of that case, which concerned contraceptive advice and treatment.^197^

Australian gender dysphoria jurisprudence has shown a patent unease with the notion that children and adolescents are *Gillick* competent to consent to gender dysphoria treatment.^198^ In *Re Alex*, discussed above, the Human Rights and Equal Opportunity Commission submitted that

a court has no power to override either the informed consent or informed refusal of a competent child to medical treatment, or, if it does have such a power, it should not as a matter of discretion exercise that power except, perhaps, in extreme circumstances.^199^

The Commission also submitted that

if this Court finds that the child has achieved ‘a sufficient understanding and intelligence’ to enable the child ‘to understand fully what is proposed’, then this Court has no further role in this matter.^200^

Without needing to determine these questions, Nicholson CJ emphasised that there was

a considerable difference between a child or young person deciding to use contraceptives as in *Gillick* and a child or young person determining upon a course that will ‘change’ his/her sex. It is highly questionable whether a 13 year old could ever be regarded as having the capacity for the latter, and this situation may well continue until the young person reaches maturity. ^201^

Bryant CJ in *Re Jamie* described medical treatment for ‘something as personal and essential as the perception of one’s gender and sexuality’ as ‘the very exemplar of when the rights of the *Gillick*-competent child should be given full effect’—yet concluded that the Family Court must determine the question of *Gillick* competence for stage two treatment.^202^ The failure to respect a finding of *Gillick* competence manifested differently in *Re Imogen*, with Watts J finding that *Gillick* competence was not determinative if parents disagree about an adolescent’s gender dysphoria diagnosis or treatment.^203^

Despite their cautious approach in gender dysphoria cases, courts have stopped short of clearly articulating this as a special category of medical treatment. In the recent UK case of *AB v CD*, the High Court stated in relation to puberty blockers that

[t]here are particular issues in relation to [puberty blockers] and there may well be justification for clinicians taking a very cautious approach in individual cases and erring on the side of having Court consideration and authorisation. However, the need for caution in imposing blanket rules, even for the most difficult categories of case, is important to have closely in mind.^204^

The Court of Appeal in *Bell* went further in forging the nexus between the nature of stage one treatment for gender dysphoria and the issue of *Gillick* competence, observing that

[t]he *ratio decidendi* of *Gillick* was that it was for doctors and not judges to decide on the capacity of a person under 16 to consent to medical treatment. Nothing about the nature or implications of the treatment with puberty blockers allows for a real distinction to be made between the consideration of contraception in *Gillick* and of puberty blockers in this case[.]^205^

It is important to note that the test for valid consent is met ‘[o]nce a patient is apprised of the broad terms of the intended procedure’.^206^ However, clinicians remain vulnerable to civil action if they do not disclose all ‘material risks’ associated with treatment for gender dysphoria in accordance with their duty to inform under the law of negligence. This possibility was highlighted by both the Court of Appeal in *Bell v Tavistock* and the High Court in *AB v CD*.^207^ The Court of Appeal in *Bell* clearly supported the capacity of *Gillick*-competent adolescents to make decisions about hormone treatment for gender dysphoria. However, it recognised ‘the difficulties and complexities associated with the question of whether children are competent to consent to the prescription of puberty blockers and cross-sex hormones’ and foreshadowed that

[g]reat care is needed to ensure that the necessary consents are properly obtained… [as] clinicians will be alive to the possibility of regulatory or civil action where, in individual cases, the issue can be tested. ^208^

In highlighting the exposure of clinicians to civil action in individual cases, the Court of Appeal has left open the door for future court involvement in gender dysphoria cases, particularly where the *Gillick* competence of an adolescent is in dispute. Our view is that, even if gender dysphoria can be described as ‘innovative’ treatment, this should not impact on an adolescent’s ability to give valid consent. Richards and Hutchison argue that although concerns around consent to innovative treatment (in relation to which healthcare providers do not know the full range of risks and benefits) are valid, ‘they are issues that arise in the context of all medical treatment’.^209^ Beattie similarly argues that deciding to take puberty blockers is no more complex than various other treatment decisions made by mature minors.^210^ Giordano and others challenge the claim that adolescents lack the capacity to give valid consent to medical treatment for gender dysphoria, arguing that complex decisions with uncertain outcomes are ‘ubiquitous’ in medicine.^211^ They suggest that

[i]t might be the case that the bar for *Gillick*-competence is more difficult to reach for very complex decisions, but this is no *a priori* reason to believe that it cannot be reached.^212^

Furthermore, as McMillan and Gavaghan point out, it is for clinicians—not courts—to decide when an adolescent has capacity to make these treatment decisions.^213^ Courts have applied a higher bar for *Gillick* competence in cases involving refusal of life-sustaining treatment and have, on occasion, overruled the views of a *Gillick*-competent child to impose treatment.^214^ This approach to refusal of treatment cases has, however, been broadly criticised as inconsistent with the principle of *Gillick* competence.^215^

The High Court in *Bell v Tavistock* held that ‘in order to achieve *Gillick* competence it is important not to set the bar too high’.^216^ The Court added that

[i]t is not appropriate to equate the matters that a clinician needs to explain, as set out in *Montgomery*, to the matters that a child needs to understand to achieve *Gillick* competence.^217^

The Court of Appeal in *Bell v Tavistock* reinforced this view, observing that

[t]he legal issue before the [High] Court was not a general inquiry into the content of information and understanding needed to secure the informed consent of a child, although we have great sympathy with the [High] Court given the large volumes of materials which informed that clinical issue.^218^

We agree that it is important not to impose too high a standard for *Gillick* competence, as the information requirements for a valid consent are different to the legal standard imposed on doctors in relation to their duty to inform patients under the law of negligence. However, the requirements for a valid consent (to prevent an action in trespass) and the duty to inform (to avoid an action in negligence), are often conflated in this debate. Confusion on this issue is potentially compounded by frequent use of the term ‘informed consent’, both in clinical practice and by courts. This term confuses the information needed for a valid consent with the information that a doctor is required to provide to a patient to discharge their duty to inform.^219^ As the High Court of Australia in *Rogers v Whitaker* pointed out

[n]othing is to be gained by reiterating… the oft-used and somewhat amorphous phrase ‘informed consent’… it is apt to mislead as it suggests a test of the validity of a patient’s consent.^220^

Although the courts have specifically warned against setting the bar for *Gillick* competence too high, our analysis of the gender dysphoria jurisprudence in Australia and the UK arguably demonstrates that a higher standard of capacity is imposed on adolescents diagnosed with gender dysphoria than that which applies to adults. This higher standard may be attributable to the specific test for *Gillick* competence as originally set out by the House of Lords, which has been interpreted as requiring the child to understand ‘all aspects of the advice’.^221^ Matthews and Smith suggest that the test outlined in *Gillick* ‘requires not merely an understanding of the decision in question, but a *full* understanding of the decision and its effects’.^222^ This interpretation of *Gillick* is controversial from a children’s rights perspective, although it has some legal underpinnings.^223^

## VI. CONCLUSION: THE NEED FOR JUDICIAL CLARITY AND TRANSPARENCY AND A ROBUST DECISION-MAKING FRAMEWORK

In this article, we have shown that, despite their involvement in the medical treatment of gender dysphoria in children and adolescents, Australian and UK courts are reluctant to engage with current clinical and ethical debate around such a treatment. In *Re Imogen*, although Watts J noted that gender dysphoria treatment is evolving, he did not see it as the Family Court’s role to explore the ‘proliferation of academic and other writings’ in recent years to make any findings about the long-term implications of current medical treatment for gender dysphoria.^224^ We consider this approach to be problematic, given that Watts J relied on the fact of disharmony amongst experts to justify the Family Court’s ongoing involvement in cases where there is a dispute or controversy. Watts J’s approach also stands in contrast to that of the Full Court in *Re Kelvin*, where the majority engaged with the current clinical evidence to depart from *Re Jamie*, noting that ‘the state of medical knowledge has evolved’,^225^ and that it was ‘readily apparent that the judicial understanding of gender dysphoria and its treatment have fallen behind the advances in medical science’.^226^

Gender dysphoria proceedings illustrate Windeyer J’s observation of the law ‘marching with medicine but in the rear and limping a little’.^227^ It is important to understand the courts’ rationale for their continued involvement in medical treatment for gender dysphoria in children and adolescents. We consider that greater transparency and clarity by courts about their role is necessary for two reasons. The first is to enable the judicial rationale to be openly critiqued and to evaluate whether court intervention is appropriate. Secondly, clarity about the basis for court intervention can help clinicians to address any genuine concerns about the current consent process. In particular, courts can provide valuable guidance about: (i) the nature of information that should be provided to adolescents and their parents (to obtain valid consent and to discharge the clinician’s duty to inform) and (ii) how that information should be provided to enhance a *Gillick*-competent adolescent’s capacity to use or weigh that information.

This article has also shown that treatment for gender dysphoria remains an area where there is often fervent disagreement, including amongst medical professionals, about the benefits and risks of treatment and long-term outcomes. It is likely that controversies and disputes will continue to arise, which will enliven the court’s jurisdiction in these cases, including potential civil action against clinicians. We argue that the courts ought to engage with this emerging and ongoing debate in individual cases.

Overall, we recommend the development of a robust clinical decision-making framework for gender dysphoria treatment, to minimise court involvement in the future, to support *Gillick*-competent adolescents to make informed decisions about their current and future health, and to contribute to the ongoing body of evidence in this area of medicine. The Australian Standards of Care are a useful starting point.^228^ The Royal Australian and New Zealand College of Psychiatrists Position Statement 103 on mental health needs of persons experiencing gender dysphoria also provides some guidance on decision-making processes.^229^ We argue elsewhere that courts should be a last resort for resolving disagreements between *Gillick-*competent adolescents and their parents over gender dysphoria treatment.^230^ We outline principles that could inform and guide the decision-making process and suggest how the law might be reformed to provide a framework that supports children and adolescents, families and clinicians in making complex decisions.^231^ Specifically, an alternative regulatory framework to the court process should promote clarity, transparency, and consistency in decision-making about treatment of gender dysphoria in children and adolescents.^232^ This could be achieved by clarifying the roles and obligations of healthcare professionals, providing ongoing training and support in the field of transgender health, implementing a regime for monitoring, recording and reporting long-term patient outcomes, and creating a process for resolving disputes that promotes reflective decision-making.^233^ This dispute resolution process should be guided by the need to respect a *Gillick*-competent adolescent’s decision to the greatest extent possible, and to explore viable options for accessing treatment. A robust decision-making framework should help to minimise disputes and the need for court involvement by addressing concerns and managing expectations for patients, their families, and the treating team.

*Conflict of interest statement*. None declared.

## Footnotes

1 In September 2021, the Family Court of Australia and the Federal Circuit Court of Australia merged to become the Federal Circuit and Family Court of Australia. In this article, we refer to the ‘Family Court’, as this was the court that heard applications for authorisation of gender dysphoria treatment pursuant to section 67ZC of the Family Law Act 1975 (Cth). This is discussed further in Section II.B.

2 On 6 April 2021, Arkansas became the first US state to criminalise medical treatment for gender dysphoria for children and adolescents, with Alabama and Tennessee proposing similar legislation; see ‘Transgender youth Treatment Banned by Arkansas’ *BBC News* (London, 6 April 2021). <www.bbc.com/news/world-us-canada-56657625> accessed 10 December 2021. For a critique of this ban on treatment, see Simon Martin, Elizabeth S Sandberg and Daniel E Shumer, ‘Criminalization of Gender-Affirming Care: Interfering with Essential Treatment for Transgender Children and Adolescents’ (2021) 385 New England Journal of Medicine 579; Abby Walch and others, ‘Proper Care of Transgender and Gender Diverse Persons in the Setting of Proposed Discrimination: A Policy Perspective’ (2021) 10 Journal of Clinical Endocrinology and Metabolism 305.

3 Steph Jowett and Fiona Kelly, ‘*Re Imogen*: A Step in the Wrong Direction’ (2021) 34 Australian Journal of Family Law 31.

4 Kirsty L Moreton, ‘A Backwards-Step for *Gillick*: Trans Children’s Inability to Consent to Treatment for Gender Dysphoria: *Quincy Bell & Mrs A v The Tavistock and Portman NHS Foundation Trust and Ors* [2020] EWHC 3274 (Admin)’ (2021) 29 Medical Law Review 699, 712.

5 [2020] FamCA 761 (*Re Imogen*).

6
*Bell v Tavistock* [2020] EWHC 3274 (Admin) (*Bell v Tavistock* (HC)) [148], [152].

7
*Re Kelvin* [2017] FamCAFC 258 [124], [134].

8 ibid; *Re Alex* [2004] FamCA 297 (*Re Alex*) [180].

9
*Bell v Tavistock* (HC) (n 6) [134].

10
*Bell v Tavistock* [2021] EWCA Civ 1363 (*Bell v Tavistock* (CoA)) [3], [35], [48], [93]. Throughout this article, we use these terms interchangeably to articulate ‘innovative treatment’, a concept that has not yet been clearly defined by courts or in the literature.

11
*Re Imogen* (n 5) [5].

12 ibid [57].

13 Georgina Dimopoulos and Michelle Taylor-Sands, ‘*Re Imogen*: The Role of the Family Court of Australia in Disputes over Gender Dysphoria Treatment’ (2021) 39(Suppl 1) Monash Bioethics Review S42.

14
*Re Alex* (n 8) [97]; *Re Alex* [2009] FamCA 1292 (*Re Alex* No 2) [14].

15 American Psychiatric Association, *Diagnostic and Statistical Manual of Mental Disorders* (5th edn, American Psychiatric Association Publishing 2013).

16 ibid.

17 Jack Turban, ‘What Is Gender Dysphoria?’ (*American Psychiatric Association*, November 2020) <www.psychiatry.org/patients-families/gender-dysphoria/what-is-gender-dysphoria> accessed 10 December 2021. The DSM-5 does not represent gender (and dysphoria) in a necessarily binary way: for example, it refers to ‘gender identity’ as ‘a category of social identity and refers to an individual’s identification as male, female, or, occasionally, *some category other than male or female*’: 451 (emphasis added). However, this has been misrepresented in gender dysphoria jurisprudence in both the UK and Australia: see, eg, *Bell v Tavistock* (HC) (n 6) [55] (the High Court of England and Wales referring to ‘transition (both medical and social) to living as the opposite sex’); *Re Alex* (n 8) [192], [196]–[197]. See also *Kevin v Attorney-General (Cth)* (2001) 165 FLR 404, 473 [315] (Chisholm J describing ‘the fundamental task of the law … in a legal and social context that divides all human beings into male and female’ as being ‘to assign individuals to one category or the other, including individuals whose characteristics are not uniformly those of one or other sex’.

18 Eli Coleman and others, *Standards of Care for the Health of Transsexual, Transgender, and Gender-Nonconforming People* (7th version, World Professional Association for Transgender Health 2012) <www.wpath.org/media/cms/Documents/SOC%20v7/SOC%20V7_English2012.pdf?_t=1613669341> accessed 17 March 2021.

19 Wylie C Hembree and others, ‘Endocrine Treatment of Gender-Dysphoric/Gender-Incongruent Persons: An Endocrine Society Clinical Practice Guideline’ (2017) 102 Journal of Clinical Endocrinology & Metabolism 3869.

20 Michelle Telfer and others, *Australian Standards of Care and Treatment Guidelines for Trans and Gender Diverse Children and Adolescents* (Version 1.3, Royal Children’s Hospital 2020) <https://www.rch.org.au/adolescent-medicine/gender-service/> accessed 17 March 2021.

21 Sari L Reisner, Asa Radix and Madeline B Deutsch, ‘Integrated and Gender-Affirming Transgender Clinical Care and Research’ (2016) 72(Suppl 3) Journal of Acquired Immune Deficiency Syndromes S235.

22 Florence Ashley, Colton M St Amand and G Nic Rider, ‘The Continuum of Informed Consent Models in Transgender Health’ (2021) 38 Family Practice 543.

23 Cassandra Spanos and others, ‘The Informed Consent Model of Care for Accessing Gender-Affirming Hormone Therapy Is Associated with High Patient Satisfaction’ (2021) 18 Journal of Sexual Medicine 201, 202.

24 Patrick Parkinson, ‘Adolescent Gender Dysphoria and the Informed Consent Model of Care’ (2021) 28 Journal of Law and Medicine 734. 

25
*Re Kelvin* (n 7) [123].

26
*Re Lucy* [2013] FamCA 518 [82].

27
*Family Law Act 1975* (Cth) s 61B.

28
*Re Tahlia* [2017] FamCA 715 [35].

29
*Re Z* (1996) 134 FLR 40 [323] (Nicholson CJ and Frederico J) (‘we see no reason to necessary limit its operation to the *parens patriae* jurisdiction’); *Jacks & Samson* [2008] FamCAFC 173 [220] (Coleman, Boland and Stevenson JJ). See also Explanatory Memorandum to the Family Law Reform Bill 1994 (Cth) [319] (that ‘[t]his jurisdiction is the *parens patriae* jurisdiction explained by the High Court in [Marion’s case]’).

30
*Re Kelvin* (n 7) [190].

31
*Re Bernadette* (2011) FamCAFC 50, [55].

32
*Re Alex* (n 8) [154].

33 A third factual issue is whether the child is currently under a care order, see *Re Alex* (n 8) [151].

34 See *Re Jamie* [2013] FamCAFC 110 [153] (Finn J) (querying ‘the usefulness of the expression “special medical procedure”’; preferring to refer to a ‘medical procedure which requires court authorisation’).

35 (1992) 175 CLR 218 (*Marion’s Case*).

36 ibid 249.

37 ibid 250.

38 ibid 249.

39
*Re Alex* (n 8). See also Marie Fox, Michael Thomson and Joshua Warburton, ‘Embodied Integrity, Shaping Surgeries and the Profoundly Disabled Child’ in Chris Dietz, Mitchell Travis and Michael Thomson (eds), *A Jurisprudence of the Body* (Palgrave Macmillan 2020) 281 (addressing the capacity of an ‘embodied integrity’ approach to protect severely disabled children from irreversible non-therapeutic bodily interventions and to frame a more appropriate ethico-legal response to their care).

40 ibid 534 [28], [180]. Cf Rachael Wallbank, ‘*Re Kevin* in Perspective’ (2004) 9 Deakin Law Review 461, 489.

41 As the condition was then known, per the fourth edition of the DSM.

42
*Re Alex* (n 8) [168].

43 ibid 537 [196].

44
*Re Jamie* (n 34).

45 ibid [106]–[107], [140(a)] (Bryant CJ), [179] (Finn J), [193] (Strickland J).

46 ibid [111] (Bryant CJ), [182] (Finn J), [195]–[196] (Strickland J).

47 ibid [136]–[137] (Bryant CJ), [186]–[188] (Finn J), [195]–[196] (Strickland J).

48
*Re Kelvin* (n 7).

49 ibid [177]–[184] (Thackray, Strickland and Murphy JJ), [226] (Ainslie-Wallace and Ryan JJ).

50 ibid [167].

51 ibid [189].

52 ibid [134].

53 See nn 6–10 above.

54
*Re Jamie* (n 34) [99].

55 See, eg, *Re Brodie (Special Medical Procedure)* [2008] FamCA 334, 230; *Re Jamie* (n 34) [99]; *Re Lucas* [2016] FamCA 1129 [3].

56
*Re Alex* (n 8).

57 [2010] FamCA 948.

58 ibid [65].

59
*Re Jamie* (n 34) [105].

60 ibid [106].

61
*Re Kelvin* (n 7) [138].

62
*Re Bernadette* [2010] FamCA 94.

63 ibid [3]–[4].

64 ibid [123].

65 ibid [79], [83], [114].

66 ibid [45], [48], [54], [59], [90], [95]. For an overview of these different treatment protocols, see Scott Leibowitz and Annelou LC de Vries, ‘Gender Dysphoria in Adolescence’ (2016) 28 International Review of Psychiatry 21; Edmond J Coleman and Walter O Bockting, *Gender Dysphoria: Interdisciplinary Approaches in Clinical Management* (Routledge 2019).

67
*Re Bernadette* (n 62) [124].

68
*Re Rae* [2017] FamCA 958 [63]. See also *Re Emery* [2016] FamCA 240 [54].

69
*Re Rae* (n 68) [57]–[58].

70
*Re Christopher* [2015] FamCA 454 [23].

71
*Re Rae* (n 68) [70]. See also *Re Jamie* (n 34) [45] (quoting the submissions of an intervener in the case which referred to a ‘lack of longitudinal studies about the long term social consequences’).

72
*Re Christopher* (n 70) [23]. In the recent Queensland Supreme Court decision for the authorisation of stage one treatment for gender dysphoria, the Court was satisfied that the proposed treatment was ‘the national and international best practice and … in accordance with the current Guidelines’, but noted that ‘the long-term impact on bone mineralisation’ of stage one treatment was ‘currently unknown’ and that the reversibility of some stage two treatments was unknown: see *Re a Declaration Regarding Medical Treatment for ‘A’* [2020] QSC 389 [37(d)], [32].

73
*Re Brodie* (n 55) [220], [223].

74
*Re Rae* (n 68) [70].

75
*Re Kelvin* (n 7) [152].

76 ibid [118].

77 ibid [205].

78 ibid [119].

79 ibid [192].

80 ibid [195].

81 ibid [148].

82 ibid [139].

83 ibid [152].

84 ibid [153]–[158].

85 ibid [159], [161].

86 ibid [159].

87 ibid [172], [175].

88 ibid [171], [172], [175], [183].

89 ibid [205].

90 ibid [116].

91
*Re Imogen* (n 5) [1], [205].

92 Three parties in addition to Imogen’s father and mother (the applicant and respondent, respectively) were involved in the hearing: an independent children’s lawyer appointed to represent Imogen’s interests, and two interveners, namely, the Commonwealth Attorney-General and the Australian Human Rights Commission.

93
*Re Imogen* (n 5) [2].

94 For a fuller critique of the *Re Imogen* decision, see Dimopoulos and Taylor-Sands (n 13); Jowett and Kelly (n 3).

95
*Re Imogen* (n 5) [35(a)].

96 ibid [35(b)].

97 ibid [35(c)].

98 ibid [35(d)].

99 ibid [5].

100 ibid [57].

101
*Re Kelvin* (n 7) [89].

102
*Re Imogen* (n 5) [57].

103 ibid.

104
*Re Kelvin* (n 7) [119]. See also *Re Lucas* (n 55) [68].

105
*Re Imogen* (n 5) [20].

106 ibid [21].

107 ibid [4], [224].

108 ibid [27].

109 ibid [154].

110 ibid [4], [230].

111 ibid [4], [226].

112 ibid [4].

113 ibid [60]–[62].

114 ibid [138].

115 ibid [137]–[139].

116 ibid [152].

117 ibid [154].

118 ibid [163].

119 ibid [162].

120 ibid [192].

121 ibid [139].

122
*Re Kelvin* (n 7) [124].

123
*Re Imogen* (n 5) [49], [59].

124 ibid [202].

125 ibid [30].

126
*Re Imogen* (n 5) [208].

127 Cf the recent decision of the High Court of Justice of England and Wales in *Bell v Tavistock* (HC) (n 6) [148], [152] (which labelled puberty-suppressing medication prescribed to adolescents diagnosed with gender dysphoria as ‘experimental’ and ‘innovative’).

128
*Re Kelvin* (n 7) [13], [124].

129
*Re Bernadette* (n 62).

130
*Re Kelvin* (n 7) [139].

131 See, eg, *Re Imogen* (n 5) [151], [162]; *Bell v Tavistock* (HC) (n 6) [22]–[25]; Tabitha Frew, Clare Watsford and Iain Walker, ‘Gender Dysphoria and Psychiatric Comorbidities in Childhood: A Systematic Review’ (2021) 73 Australian Journal of Psychology 255; Juan Carlos d’Abrera and others, ‘Informed Consent and Childhood Gender Dysphoria: Emerging Complexities in Diagnosis and Treatment’ (2020) 28 Australasian Psychiatry 536; Jack Drescher and Jack Pula, ‘Ethical Issues Raised by the Treatment of Gender-Variant Prepubescent Children’ (2014) 44 LGBT Bioethics: Visibility, Disparities, and Dialogue S17; Ada S Cheung and others, ‘Position Statement on the Hormonal Management of Adult Transgender and Gender Diverse Individuals’ (2019) 211 Medical Journal of Australia 127.

132
*Bell v Tavistock* (HC) (n 6).

133 ibid.

134 ibid [134].

135 ibid [135].

136 Peter Dunne, ‘Case Comment: Childhood in Transition: Can Transgender and Non-Binary Minors Provide Lawful Consent to Puberty Blockers?’ (2021) 80 Cambridge Law Journal 15.

137
*AB v* CD & Ors [2021] EWHC 741 (Fam) (*AB v CD*).

138 ibid [97].

139
*Bell v Tavistock* (CoA) (n 10) [3].

140 ibid.

141 ‘We’ve Won: Bell v Tavistock Judgment Quashed by Court of Appeal’ (*Good Law Project*, 17 September 2021) <https://goodlawproject.org/update/weve-won-bell-v-tavistock/> accessed 10 December 2021.

142 ‘Court of Appeal Ruling Step Forwards for Trans Rights’ (*Liberty*, 17 September 2021) <www.libertyhumanrights.org.uk/issue/liberty-court-of-appeal-ruling-step-forwards-for-trans-rights/#:~:text=Liberty%20has%20welcomed%20the%20Court , affirming%20and%20a%20human%20right> accessed 10 December 2021.

143 John McMillan and Colin Gavaghan, ‘Mature Minors and Gender Dysphoria: A Matter for Clinicians Not Courts’ (2021) 47 Journal of Medical Ethics 717, 717.

144 Annelou LC de Vries and others, ‘Bell v Tavistock and Portman NHS Foundation Trust [2020] EWHC 3274: Weighing Current Knowledge and Uncertainties in Decisions about Gender-Related Treatment for Transgender Adolescents’ (2021) 22 International Journal of Transgender Health 217, 217.

145
*Bell v Tavistock* (CoA) (n 10) [92]. See also discussion in Section V.D.

146 Bernadette Richards and Katrina Hutchison, ‘Consent to Innovative Treatment: No Need for a New Legal Test’ (2016) 23 Journal of Law and Medicine 938; Tina Cockburn and Michael Fay, ‘Consent to Innovative Treatment’ (2019) 11 Law, Innovation and Technology 34.

147 For a fuller discussion of this point in the context of gender dysphoria treatment, see Georgina Dimopoulos, *Decisional Privacy and the Rights of the Child* (Routledge, forthcoming 2022) ch 4. See also Brendan S Abel, ‘Hormone Treatment of Children and Adolescents with Gender Dysphoria: An Ethical Analysis’ (2014) 44 (Suppl 4) *Hastings Centre Report* S2.

148
*Bell v Tavistock* (HC) (n 6) [139].

149 Ibid [144].

150 See, eg, Joel Feinberg, ‘The Child’s Right to an Open Future’ in William Aitken and Hugh La Follette (eds), *Whose Child? Children’s Rights, Parental Authority, and State Power* (Rowman and Littlefield 1980) 124.

151
*Bell v Tavistock* (CoA) (n 10) [29].

152 See discussion in Section IV.B above.

153
*AB v CD* (n 137) [122].

154 ibid.

155 See, eg, *Bell v Tavistock* (HC) (n 6) [134].

156 Lauren Notini, Rosalind McDougall and Ken C Pang, ‘Should Parental Refusal of Puberty-Blocking Treatment be Overridden? The Role of the Harm Principle’ (2019) 29 American Journal of Bioethics 69, 70; Mike O’Connor and Bill Madden, ‘In the Footsteps of Teiresias: Treatment for Gender Dysphoria in Children and the Role of the Courts’ (2019) 27 Journal of Law and Medicine 149, 158–61.

157 Chloë De Roo and others, ‘Fertility Options in Transgender People’ (2016) 28 International Review of Psychiatry 112; Eva Feigerlová and others, ‘Fertility Desires and Reproductive Needs of Transgender People: Challenges Considerations for Clinical Practice’ (2019) 91 Clinical Endocrinology 10.

158 Sandra Duffy, ‘Latest English Court Ruling on Puberty Blockers Is a Step Forward’ *Scottish Legal News* (Scotland, 31 March 2021) < www.scottishlegal.com/article/dr-sandra-duffy-latest-english-court-ruling-on-puberty-blockers-is-step-forward> accessed 10 December 2021.

159 Royal Australian and New Zealand College of Psychiatrists, ‘Position Statement 103: Recognising and Addressing the Mental Health Needs of People Experiencing Gender Dysphoria/Gender Incongruence’ (*Royal Australian**and**New Zealand College of Psychiatrists*, August 2021) < https://www.ranzcp.org/news-policy/policy-and-advocacy/position-statements/gender-dysphoria> accessed 10 December 2021.

160 Cheung and others, ‘Position Statement on the Hormonal Management of Adult Transgender and Gender Diverse Individuals’ (2019) 211 Medical Journal of Australia 127, 127.

161 Dominic Wilkinson and Julian Savulescu, ‘Puberty-Blocking Drugs: The Difficulties of Conducting Ethical Research’ *The Conversation* (Australia, 26 July 2019) < https://theconversation.com/puberty-blocking-drugs-the-difficulties-of-conducting-ethical-research-120906> accessed 10 December 2021.

162
*Re Kelvin* (n 7) [17]–[23], [161].

163 See Telfer and others (n 20) 5; See also Peggy T Cohen-Kettenis, Thomas D Steensma and Annelou LC De Vries, ‘Treatment of Adolescents with Gender Dysphoria in the Netherlands’ (2011) 20 Child and Adolescent Psychiatric Clinics of North America 689; Stephen Feder and others, ‘Exploring the Association Between Eating Disorders and Gender Dysphoria in Youth’ (2017) 25 Journal of Treatment and Prevention 310.

164
*Bell v Tavistock* (HC) (n 6) [135].

165 ibid [134].

166 Edmund Horowicz, ‘Rethinking “Need” for Clinical Support in Transgender and Gender Non-Conforming Children Without Clinical Classification: Learning from “the Paper I Almost Wrote”’ (2021) 35 Bioethics 246.

167 ibid.

168 Heather Brunskell-Evans, ‘The Medico-Legal “Making” of “The Transgender Child”’ (2019) 27 Medical Law Review 640.

169
*Re Imogen* (n 5); *Bell v Tavistock* (HC) (n 6) [134]; *AB v CD* (n 137) [97].

170 See discussion in Section IV.B.

171
*Bell v Tavistock* (HC) (n 6) [136].

172 Moreton (n 4) 712. See also *Re Jamie* (n 34) [108]; *Re Kelvin* (n 7), discussed in Section II.B above.

173
*Bell v Tavistock* (CoA) (n 10) [92].

174 Moreton (n 4) 706.

175
*Simms v Simms* [2002] EWHC 2734 (Fam) [57]. See also *University College London Hospitals v KG* [2018] EWCOP 29.

176
*AB v CD* (n 137) [97], [99]. For critique of this analysis see Moreton (n 4) 706.

177
*R (Burke) v General Medical Council* [2005] EWHC 1879 (Admin) [28].

178
*B v D* [2017] EWCOP 347 [58].

179 For a brief history of the concept of ‘innovative practice’ in medicine, see Jake Earl, ‘The Belmont Report and Innovative Practice’ (2020) 63 Perspectives in Biology and Medicine 313, 315–18.

180 National Commission for the Protection of Human Subjects of Biomedical and Behavioral Research, *The Belmont Report: Ethical Principles and Guidelines for the Protection of Human Subjects of Research* (Department of Health Education, and Welfare 1978) 3–4.

181 ibid.

182 Earl (n 179) 8.

183 ibid.

184 Joe Brierly and Vic Larcher, ‘Compassionate and Innovative Treatments in Children: A Proposal for an Ethical Framework’ (2009) 94 Archives of Disease in Childhood 651, 654.

185 Tsachi Keren-Paz, Tina Cockburn and Alicia El Hajc, ‘Regulating Innovative Treatments: Information, Risk Allocation and Redress’ (2019) 11 Law, Innovation and Technology 1, 1 (emphasis in original).

186 Cockburn and Fay (n 146) 45.

187 Jean V McHale, ‘Falling Between the Gaps Post the Declaration of Helsinki: Innovative Medical Treatment in England: The Case for Comprehensive Legal Regulation’ (2019) Law, Innovation and Technology 93, 97, citing Nayha Sethi, ‘Regulating for Uncertainty: Bridging Blurred Boundaries in Medical Innovation, Research and Treatment’ (2019) 11 Law, Innovation and Technology 112.

188 McHale (n 187) 97.

189 O’Connor and Madden (n 156) 162–63.

190 Moreton (n 4).

191 See, eg, Keren-Paz, Cockburn and El Hajc (n 185) and references cited therein.

192 Cockburn and Fay (n 146) 42. See also Jonathan Montgomery, ‘The “Tragedy” of Charlie Gard: A Case Study for Regulation of Innovation?’ (2019) 11 Law, Innovation and Technology 155, 161–62 (referring to a ‘tension between science and the demand for unproven therapies’ and ‘risks of over-optimism on the part of patients’).

193
*Marion’s Case* (n 35) 250.

194
*Re Jamie* (n 34); *Re Kelvin* (n 7); *Re Matthew* [2018] FamCA 161.

195 Laura L Kimberly and others, ‘Ethical Issues in Gender-Affirming Care for Youth’ (2018) 142 Pediatrics 1, 4.

196
*Gillick v West Norfolk and Wisbech Area Health Authority* [1986] AC 112 (HL), 188–89 (Lord Scarman). See also *Marion’s Case* (n 35) 237–38 (emphasis added).

197 Lisa Young, ‘Mature Minors and Parenting Disputes Australia: Engaging with the Debate Best Interests v Autonomy’ 42 UNSW Law Journal 1362, 1364; Jowett and Kelly (n 3) 46.

198 Dimopoulos and Taylor-Sands (n 13); Young (n 197); Jowett and Kelly (n 3).

199
*Re Alex* (n 8) [170].

200 ibid [172].

201 ibid [173].

202
*Re Jamie* (n 34) [135].

203
*Re Imogen* (n 5) [35(c)].

204
*AB* *v* *CD* (n 137) [99].

205
*Bell v Tavistock* (CoA) (n 10) [76].

206 Cockburn and Fay (n 146) 41.

207
*Bell v Tavistock* (CoA) (n 10) [92]; *AB v CD* (n 137) [127].

208
*Bell v Tavistock* ibid.

209 Richards and Hutchison (n 146) 938.

210 Cameron Beattie, ‘High Court Should Not Restrict Access to Puberty Blockers for Minors’ (2021) Journal of Medical Ethics (online first). <https://dx.doi.org/10.1136/medethics-2020-107055> accessed 7 June 2022.

211 Simona Giordano, Fae Garland and Søren Holm, ‘Gender Dysphoria in Adolescents: Can Adolescents or Parents Give Valid Consent to Puberty Blockers’ (2021) 47 Journal of Medical Ethics 324.

212 ibid.

213 McMillan and Gavaghan (n 143) 717.

214 See, eg, *Minister for Health v AD* [2004] WASC 286; *X v The Sydney Children’s Hospital Network* (2013) 85 NSWLR 294.

215 For a detailed discussion of refusal of treatment cases, which is beyond the scope of this article, see Gillian Douglas, ‘The Retreat from *Gillick*’ (1992) 55 Modern Law Review 569; Leanne Bunney, ‘The Capacity of Competent Minors to Consent to and Refuse Medical Treatment’ (1997) 5 Journal of Law and Medicine 52; Nicholas J Lennings, ‘Forward, *Gillick*: Are Competent Children Autonomous Medical Decision Makers? New Developments in Australia’ (2015) 2 Journal of Law and Biosciences 459.

216
*Bell v Tavistock* (HC) (n 6) [130].

217 ibid. The UK Supreme Court held in *Montgomery* that doctors must disclose all material risks involved in any recommended treatment to discharge their duty to inform to adult patients: *Montgomery v Lanarkshire Health Board* [2015] UKSC 11, [2015] 2 WLR 768.

218
*Bell v Tavistock* (CoA) (n 10) [79].

219 See Cockburn and Fay (n 146) 37 (noting that ‘to defeat a trespass claim consent must be to the broad nature of the intended treatment, not the risks and implications inherent in the procedure’).

220
*Rogers v Whitaker* (1992) 175 CLR 479, 490.

221
*Axon v The Secretary of State for Health* [2006] EWHC (Admin) 37, [2006] 2 WLR 1130 [154] (Silber J).

222 Ben Matthews and Malcolm Smith, ‘Children and Consent to Medical Treatment’ in Ben White, Fiona McDonald and Lindy Willmott (eds), *Health Law in Australia* (3rd edn, Thomson Reuters 2018) 192, 194.

223 For a critique of the stricter test for Gillick-competence, see Jamie Potter, ‘Rewriting the Competency Rules for Children: Full Recognition of the Young Person as Rights-Bearer’ (2006) 14 Journal of Law and Medicine 64, 68–69; Michael Freeman, ‘Rethinking *Gillick*’ International Journal of Children’s Rights, 13, pp. 201–17’ in Andrew Bainham (ed), *Parents and Children* (Routledge 2008).

224
*Re Imogen* (n 5) [57].

225
*Re Kelvin* (n 7) [159].

226 ibid [152].

227
*Mount Isa Mines Pty Ltd v Pusey* (1970) 125 CLR 383, 395.

228 Telfer and others (n 20).

229 Royal Australian & New Zealand College of Psychiatrists (n 159).

230 Dimopoulos and Taylor-Sands (n 13).

231 ibid.

232 Ibid.

233 Ibid.

